# Combined IL-21 and Low-Dose IL-2 therapy induces anti-tumor immunity and long-term curative effects in a murine melanoma tumor model

**DOI:** 10.1186/1479-5876-4-24

**Published:** 2006-06-13

**Authors:** Hong He, Preya Wisner, Guojun Yang, Hong-Ming Hu, Dan Haley, William Miller, Aisling O'Hara, W Gregory Alvord, Christopher H Clegg, Bernard A Fox, Walter J Urba, Edwin B Walker

**Affiliations:** 1 Robert W. Franz Cancer Research Center, Earle A. Chiles Research Institute, Providence Portland Medical Center, Portland OR, USA; 2 DMS-National Cancer Institute, Frederick MD, USA; 3 Immunology Research, ZymoGenetics, Seattle WA, USA

## Abstract

**Background:**

In vivo studies have recently demonstrated that interleukin 21 (IL-21) enhances the anti-tumor function of T-cells and NK cells in murine tumor models, and the combined use of IL-21 and IL-15 has resulted in prolonged tumor regression and survival in mice with previously established tumors. However, the combined anti-tumor effects of IL-21 and low dose IL-2 have not been studied even though IL-2 has been approved for human use, and, at low dose administration, stimulates the proliferation of memory T cells, and does not significantly increase antigen-induced apoptosis or regulatory T cell (Treg) expansion. This study examined whether recombinant IL-21 alone or in combination with low-dose IL-2 could improve the in vivo anti-tumor function of naïve, tumor-antigen specific CD8^+ ^T cells in a gp100_25–33 _T cell receptor transgenic pmel murine melanoma model.

**Methods:**

Congenic C57BL/6 (Ly5.2) mice bearing subcutaneous B16F10 melanoma tumors were sublethally irradiated to induce lymphopenia. After irradiation naive pmel splenocytes were adoptively transferred, and mice were immunized with bone marrow-derived dendritic cells pulsed with human gp100_25–33 _(hgp100_25–33_). Seven days after vaccination groups of mice received 5 consecutive days of intraperitoneal administration of IL-2 alone (20 × 10^3 ^IU), IL-21 alone (20 μg) or IL-21 and IL-2. Control animals received no cytokine therapy.

**Results:**

IL-21 alone and IL-2 alone both delayed tumor progression, but only IL-21 significantly augmented long-term survival (20%) compared to the control group. However, combination therapy with IL-21 and IL-2 resulted in the highest long-term (>150 days) tumor-free survival frequency of 46%. Animals that were tumor-free for > 150 days demonstrated tumor-specific protection after rechallenge with B16F10 melanoma cells. At peak expansion (21 days post vaccination), the combination of IL-21 plus IL-2 resulted in a 2- to 3-fold higher absolute number of circulating tumor antigen-specific pmel CD8^+ ^T cells than was stimulated by IL-2 or IL-21 alone. Pmel CD8^+ ^T cells were predominantly partitioned into central memory (CD62L^+^/CD127^+^) or effector-memory (CD62L^-^/CD127^+^) phenotypes by day 28-post vaccination in IL-21 + IL-2 treated mice.

**Conclusion:**

These observations support the potential use of IL-21 and low-dose IL-2 therapy in combination with a tumor-antigen vaccine and lymphopenic conditioning in future cancer clinical trials to maintain high numbers of anti-tumor memory CD8^+ ^T cells with the potential to sustain long term tumor regression and survival.

## Background

IL-21 has potent immunomodulatory effects on T cells and NK cells [1]. However, the current understanding of the effects of IL-21 on the regulation of both antigen-independent homeostatic and antigen-stimulated proliferation and activation of naive and memory CD8^+ ^T cells is confounded by conflicting data. IL-21 has been reported both to synergize with IL-15 to increase homeostatic antigen-independent proliferation of naive and memory CD8^+ ^T cells [2,3], and to inhibit IL-15 induced homeostatic proliferation of memory T cells [4]. While evidence suggests IL-21 enhances primary antigen-stimulated T-cell proliferation and functional activation [3-6], there is disagreement over whether this effect occurs during priming [5], or during the late expansion phase of antigen-specific proliferation [6]. Recent data suggests IL-21 primarily increases the frequency and absolute number of in vitro stimulated antigen-specific CD8^+ ^T cells by enhancing the rate of proliferation rather than by reducing apoptosis [5]. Alternatively, other studies indicate IL-21 maintains increased numbers of antigen-stimulated effector and long-term memory CD8^+ ^T cells by reducing apoptosis while maintaining a low rate of cell division [6]. These conflicting data may be attributed to differences in the in vitro and in vivo models employed in the individual studies. However, they suggest that much work remains in the effort to elucidate the mechanism of IL-21 regulation of T-cell immune responses, including any potential role it may play in the regulation of T-cell mediated anti-tumor immunity through the induction of functionally active tumor-specific effector and memory T cells (Reviewed in [1]).

In vivo data demonstrate that IL-21 enhances the anti-tumor function of T-cells and NK cells. Several studies have shown that genetically engineered IL-21-secreting murine tumors activate potent NK and CD8^+ ^T cell mediated anti-tumor responses [7-9]. IL-21 was also compared to IL-2 and IL-15 injected intraperitoneally (IP) subsequent to challenge with the ova-expressing E.G7 thymoma in either naive C57BL/6 mice, or in mice infused with OT-1 transgenic CD8^+ ^T cells [6]. In both models, IL-2 and IL-15 delayed tumor growth, but only IL-21 resulted in significant prevention of tumor progression and improved survival beyond 50 days from tumor challenge. The combination of IL-21 and IL-15 was also administered IP subsequent to human gp100_25–33 _(hgp100_25–33_) peptide vaccination of lymphopenic, tumor-bearing (B16F10 melanoma) C57BL/6 mice [3]. Before vaccination mice received in vitro stimulated (IVS) pmel transgenic (Tg) CD8^+ ^T cells, which have a T-cell receptor specific for the murine H-2D^b ^restricted gp100_25–33 _peptide of the B16F10 melanoma associated gp100 protein, and strongly cross react with hgp100_25–33 _[10]. The combination of IL-21 + IL-15 resulted in prolonged tumor regression and survival out to 32 days. Mice treated with the vaccine plus IL-15 or IL-21 alone all died of tumor within 32 days of treatment. No data was presented demonstrating long-term survival (>60 days) of mice treated with IL-21 + IL-15. Notably, IL-2 was not tested in vivo in combination with IL-21 in this study primarily because the combination of these two cytokines failed to drive antigen-independent homeostatic proliferation of murine CD8^+ ^splenocytes in vitro [3], and the concern that high-dose IL-2 has been shown to decrease memory CD8^+ ^T-cell function by inducing regulatory T-cells and increasing antigen-driven apoptosis [11-14]. However, other data have demonstrated that the in vitro maintenance of antigen-stimulated murine T cells in low-dose IL-2 resulted in the proliferative expansion of cells with a memory phenotype similar to that induced by IL-15; these cells were capable of long-term survival in vivo, and potent proliferative and functional responses after secondary in vivo challenge [15]. Thus, treatment with IL-21 + low-dose IL-2 after the adoptive transfer of pmel Tg CD8^+ ^T cells into mice with established B16 melanoma tumors (pmel Tg/B16 model) might yield anti-tumor effects comparable to or greater than those observed with IL-21+IL-15.

The pmel Tg/B16 melanoma model has been employed repeatedly in recent studies to model immunotherapeutic strategies for treatment of pre-existing disease. Most of these experiments have focused on the adoptive transfer of antigen-educated pmel CD8^+ ^splenocytes stimulated in vitro with hgp100_25–33 _peptide and IL-2, and the subsequent in vivo expansion of these highly activated cells in tumor-bearing mice with hgp100_25–33 _vaccination and follow-on cytokine therapy [3,16-18]. While this strategy has resulted in significant tumor regression, little data has been presented demonstrating long-term curative effects (≥ 100 days) with IL-2 alone [16,17], IL-15 alone [18], or the combination of IL-15 and IL-21 [3] – except in mice with small tumors (≤ 10 mm^2^) at the time of treatment, or after the adoptive transfer of very large numbers (10^7^) of IVS pmel CD8^+ ^T cells [16]. The adoptive transfer of highly activated IVS T-cells may not produce long-term anti-tumor curative effects due to functional and proliferative exhaustion [19-21]. This conclusion is supported by a series of provocative experiments using the pmel Tg/B16 model in which the adoptive transfer of naive or "early" IVS-effector pmel CD8^+ ^T cells into lymphopenic tumor-bearing recipient mice resulted in much more durable tumor regression following hgp100_25–33 _vaccination and IL-2 therapy compared to the poor therapeutic effects observed using IVS expanded, highly stimulated intermediate or late-stage effector T cells [22]. Thus, as described herein, the adoptive transfer of naïve, tumor antigen-specific CD8^+ ^T cells in this model prior to vaccine and IL-21 + low-dose IL-2 therapy may provide a better opportunity to study methods of generating both effector and long-term anti-tumor memory T-cell function than can be achieved with highly activated IVS T cells.

There have been few published studies describing the anti-tumor effects of recombinant IL-21 in conjunction with hgp100_25–33 _vaccination in B16F10 tumor-bearing lymphopenic mice receiving naive pmel Tg CD8^+ ^T cells. There have, to our knowledge, been no reports describing the potential synergy of IL-21 + low-dose IL-2 in this or other tumor models. Herein we describe the long-term curative effect of combined IL-21 + low-dose IL-2 cytokine therapy in lymphopenic, tumor-bearing C57BL/6 mice infused with naive pmel splenocytes and vaccinated with the hgp100_25–33 _melanoma peptide.

## Materials and methods

### Mice

Congenic C57BL/6 (Ly5.2) mice (Charles River Laboratories, Inc. NCI-Frederick) bred at the Earle A. Chiles Research Institute (EACRI) served as recipient mice for tumor inoculation and pmel Tg CD8^+ ^T-cell adoptive transfer in all experiments. Pmel Tg mice express a TCR specific for an H-2D^b ^epitope (gp100_25–33_) of the melanoma-associated gp100 protein on the C57BL/6 (Ly5.1) background [10,16]. Virtually all (>90%) CD8^+^T cells in pmel Tg mice were Vβ13^+^, and were also distinguishable by a monoclonal antibody specific for a point mutation of the CD45 epitope (CD45.2). Pmel Tg mice were the generous gift of Dr. Nicholas Restifo (Surgery Branch, NCI-NIH) and were bred at EACRI. Male or female mice (10–16 weeks) were used in separate experiments. All mice were treated according to the regulations and guidelines of the Institutional Animal Care and Use Committee.

### Tumor cell lines, tumor inoculation and in vivo measurement

The B16 F10 melanoma and 3LL (Lewis Lung) cell lines were obtained from ATCC. Both cell lines were maintained in complete medium consisting of RPMI 1640 supplemented with 10% heat-inactivated fetal bovine serum (Biofluids, Rockville, MD), 0.03% L-glutamine, 100 μg/ml streptomycin, 100 μg/ml penicillin, 50 μg/ml gentamicin sulfate and 50 mmol 2-mercaptoethanol. Tumor was established by injecting mice subcutaneously in the flank with 2 × 10^5 ^B16 F10 melanoma cells in 0.1 ml of phosphate buffered balanced salt solution (PBS). In tumor rechallenge experiments mice were injected subcutaneously in the opposite flank with 5.0 × 10^5 ^B16 F10 melanoma cells or 5.0 × 10^4 ^3LL tumor cells in 0.1 ml of PBS. Tumor growth was monitored three times a week by measurement of two perpendicular diameters using a digital caliper. The products of the perpendicular diameters plotted for multiple animals are presented as the mean mm^2 ^± SEM. Mice were sacrificed when tumors exceeded 200 mm^2^. Survival was analyzed by using Kaplan-Meier statistics.

### Adoptive cell transfer

Splenocytes from pmel transgenic mice were depleted of erythrocytes with ACK lysing buffer, and 4 × 10^6 ^were adoptively transferred via I.V. injection into C57BL/6 (Ly5.2) mice. Pmel spleen cells contained on average 20%–25% (~1 × 10^6^) naive CD45.2^+ ^CD8^+ ^T cells.

### Peptides

The H-2D^b^-restricted hgp100_25–33 _peptide, (KVPRNQDWL) was used as the CD8^+ ^immunogen [16], and the I-A^b^-restricted epitope of Plasmodium falciparum (NANPNVDPNANP), hereafter referred to as "NV", was used as a CD4^+^directed helper peptide [23,24]in all experiments. The peptides were made by Invitrogen Inc. and purified by reverse-phase high-performance liquid chromatography. Purity of >99% was confirmed by mass spectrometry.

### Preparation of dendritic cells

Bone marrow-derived murine dendritic cells (DCs) were generated as described previously [25]. Briefly, cells from the femur of C57BL/6 mice were grown at 1 × 10^6 ^cells/ml in RPMI 1640 complete medium supplemented with 25 ng/ml of murine GM-CSF (PeproTech, Rocky Hill, NJ). Fresh medium supplemented with GM-CSF was added on day 3, and all loosely adherent cells were transferred to petri dishes on day 6. Three days later, nonadherent and loosely adherent cells were harvested, washed, and frozen in 10% DMSO and 90% FCS in liquid nitrogen at 10^7 ^cells per vial. Frozen DCs were thawed and pulsed for 2 hours at 37°C with hgp100_25–33 _(1 μg/ml), and 1 μg/ml of the NV peptide in complete medium. DCs were washed three times with PBS before injection. DC purity and maturation were confirmed by flow cytometry analysis after staining with antibodies against MHC class I (H-2D^b^, H-2K^b^), class II (I-A^b^), CD11c, CD40, CD80, CD86. (BD Biosciences PharMingen, San Diego, CA.)

### Vaccine and cytokine therapy regimen

After B16F10 tumor cells were injected subcutaneously on day 0, groups of 5–10 mice per test group were sublethally irradiated (500 cGy) 7 days later to induce lymphopenia. All animals were then infused IV with 4 × 10^6 ^pmel splenocytes, which equated to the transfer of 8 × 10^5 ^- 1 × 10^6 ^naive CD45.2^+^/CD8^+ ^pmel T cells. Immediately after cell transfer, mice were immunized subcutaneously with 2 × 10^6 ^C57BL/6 DCs pulsed with hgp100_25–33 _(1 μg/ml) and NV peptide (1 μg/ml). The hgp100_25–33 _and NV peptide pulsed DC immunization was the standard vaccine in all experiments. Seven days after pmel T cell transfer and vaccination, mice received 5 consecutive days (days 14–18 post tumor inoculation) of IP injection of recombinant human IL-2 at 20 × 10^3 ^IU/dose (Chiron Corporation, Emoryville, CA), murine IL-21 at 20 μg/dose (ZymoGenetics Corporation, Seattle, WA), or the combination of IL-2 and IL-21 at the same dosage used for single cytokine injections.

### Flow cytometry

Except where noted, cell surface staining of peripheral blood lymphocytes and spleen cells was performed using BD Biosciences PharMingen (San Diego, CA) reagents. Single-cell suspensions were incubated with anti-mouse CD16/32 (eBioscience, San Diego, CA) to block Fc receptors. Cells were then stained with allophycocyanin (APC) conjugated anti-CD8 and fluorescein isothiocyanate (FITC)-labeled anti-CD45.2, phycoerythrin (PE) anti-CD62L and PE-Cy7 anti-CD127 (eBiosciences). Data were acquired on a FACS Calibur (BD Biosciences, San Jose, CA.) and analyzed using Cellquest Pro software (version 4.0.1). Peripheral blood and tissue derived-lymphocytes were analyzed by direct ex vivo interrogation of fresh samples. The frequency of pmel T cells was determined by measuring the frequency of total gated CD8^+ ^T cells, which were also CD45.2^+^. CD8^+ ^T cells from recipient Ly5.2 mice could be distinguished from donor pmel CD8^+ ^T-cells since recipient T cells did not express the CD45.2 point mutation, and thus were not stained with the anti-CD45.2 antibody. "Flow-Count" fluorospheres (Beckman-Coulter; Miami, Fl.) were added to each sample before event acquisition to determine the absolute cell count according to the manufacturer's instructions. A minimum of 8000 "Flow Count" beads were counted for each sample collected. The absolute number of pmel CD8^+ ^T cells per μl of blood was determined by first calculating the ratio of beads counted by flow divided by the total number of beads added to the sample, and multiplying this ratio times the known volume of the test sample of blood; this determined the fraction of the total test sample volume (in μls) analyzed. The total sample volume analyzed (in μls) was then divided into the total analysis pmel cell count to give pmel^+^CD8^+ ^T cells/μl.

Cytokine flow cytometry (CFC) analysis was performed by stimulating 10^6 ^spleen cells suspended in 250 ul of complete medium in a microtiter well (96 well plate) with hgp100_25–33 _peptide (1 ug/ml). Splenocytes were cultured with antigen and brefeldin A (10 ug/ml) for 5 hours at 37°C. Cells were washed twice in PBS and incubated with anti-mouse CD16/32 (FcR-blocking) monoclonal antibody (eBiosciences). Cells were then stained with APC-conjugated CD8 and FITC-labeled CD45.2 (eBiosciences) antibodies for 30 minutes at 4°C, washed 2× in PBS, and fixed and permeablized with 100 ul Cytofix/Cytoperm™ buffer/well (BD Biosciences Pharmingen) for15 minutes at room temperature. Cells were washed 2× in PermWash™ buffer (BD Biosciences Pharmingen) and resuspended in 100 ul of PermWash™ buffer. PE-conjugated anti-IL-2, IFNγ or TNFγ specific antibodies (BD Biosciences Pharmingen) were added at optimal dilution and incubated for one hour at 4°C; PE-conjugated isotype controls were used to determine non-specific cytoplasmic background staining. Cells were washed 2× with PBS and analyzed by flow cytometry. Cytokine positive cell frequencies were determined for pre-gated CD8^+^/CD45.2^+^pmel T cells.

### Statistical analysis

Phenotype data in this study were analyzed using repeated measures analysis of variance (ANOVA), analysis of covariance (ANCOVA), linear hierarchical mixed-effects regression models, nonparametric (distribution free) tumor growth analyses, and simple and advanced graphical techniques [26-28]. Animal survival data were analyzed with standard log-rank statistics and Kaplan-Meier plots. Pair wise *a posteriori *comparisons among treatment conditions were performed with standard post hoc tests (i.e. Tukey's test). Interpretations of tumor growth results from follow-up tests were consistent with those obtained from global analyses. Hence, for simplicity we report probability (p) values obtained from follow-up nonparametric Wilcoxon tests at specific time points. All tests were two-sided; probability values less than 0.05 were considered significant.

## Results

### Combined IL-21 + IL-2 therapy significantly enhanced anti-tumor immunity

**Figure 1:**
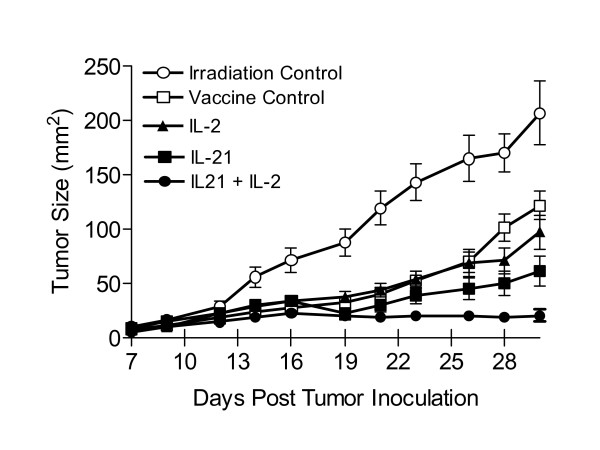
B16F10 melanoma tumor growth in hgp100_25–33 _vaccinated and cytokine treated lymphopenic mice. Tumor size is presented as the mean (mm^2^) ± SEM of the cumulative analysis of replicate mice (≥ 15) for each test group from 3 separate experiments. Cytokines were administered for 5 consecutive days beginning on day 14 when the average tumor size for vaccinated mice from all test groups in 3 experiments was 26 mm^2 ^± 2.4 mm (N = 73 mice). By day 30 IL-21 + low-dose IL-2 treated mice showed significant inhibition of tumor growth compared to the vaccine control (p < 0.0001), or compared to vaccinated mice treated with IL-2 (p = 0.0002) or IL-21 only (p = 0.0024).

Seven days after subcutaneous injection of 2 × 10^5 ^B16F10 melanoma cells C57BL/6 mice were sublethally irradiated, infused with naive pmel splenocytes and vaccinated with hgp100_25–33 _pulsed DCs. Seven days later therapy was initiated with 5 daily IP injections (days 14–18 post tumor inoculation) of IL-2 alone, IL-21 alone or both cytokines. The optimal dose of IL-21 (20 μg/dose) was established previously [6]; and the optimal IL-2 dose (20 × 10^3 ^IU/injection) was established by titration (data not shown). Figure 1 shows the cumulative mean tumor size from three separate experiments during the first four weeks following tumor inoculation (≥ 15 mice/group). On day 14, just prior to cytokine administration, the mean tumor size for all vaccinated mice (N = 73) was 26 mm^2 ^(± 2.4 mm). Thus, all vaccinated mice had established disease prior to cytokine therapy. Global analysis showed that each therapy, including the vaccine alone, reduced the rate of tumor growth compared to the irradiation only control. By day 21, vaccination combined with IL-21 + IL-2 treatment inhibited tumor growth significantly better than vaccination alone (p = 0.0013) and vaccination combined with IL-2 (p = 0.0044) or with IL-21 (p = 0.044). By day 28, vaccination combined with IL-21+ IL-2 again inhibited tumor growth significantly better than vaccination alone (p < 0.0001) and vaccination combined with IL-2 (p = 0.0006) or with IL-21 (p = 0.0033). By day 30, vaccination combined with IL-21 + IL-2 similarly inhibited tumor growth significantly better than vaccination alone (P < 0.0001), but produced higher statistically significant inhibition than vaccination combined with IL-2 (p = 0.0002) or with IL-21 (p = 0.0024) when compared to day 28. Mice in the irradiation only (lymphopenic) control group exhibited rapid tumor growth; the mean tumor size for all animals reached > 200 mm^2 ^30 days after tumor inoculation. Similarly, unvaccinated tumor-bearing control mice which were irradiated and treated with IL-21 or IL-21 + IL-2 only had comparable rapid tumor growth (data not shown). Mice treated with IL-21 + IL-2 after vaccination were the only animals that experienced no significant increase in tumor size (Figure 1).

### IL-21 + IL-2 improved long-term tumor-free survival

**Figure 2:**
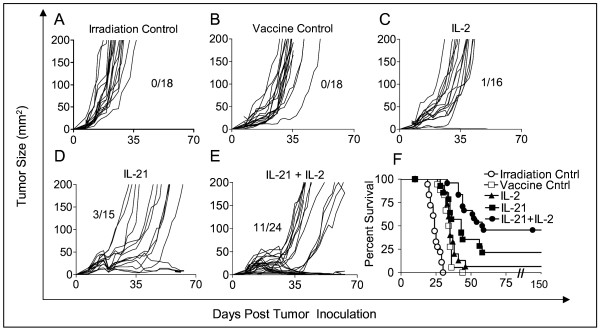
Rate of tumor growth and long-term survival in tumor-bearing mice after hgp100_25–33 _vaccination and cytokine therapy. Cumulative analysis of tumor growth rates are depicted for all replicate mice from 3 separate experiments in which mice were irradiated only (A), irradiated and vaccinated (B), or irradiated, vaccinated and treated with IL-2 (C), IL-21 (D), or IL-21 + IL-2 (E). Both IL-21 and IL-21 + IL-2 therapy delayed tumor progression in >50% of treated mice until day 42 while tumors in control and IL-2 treated mice all progressed more rapidly. IL-21 + IL-2 treated mice had a significant survival advantage (46%) over IL-21 (p = 0.035), or IL-2 (p << 0.0001) treated mice; and IL-21 therapy alone (20% survival) was significantly better than the vaccine only (p = 0.03) (F).

Having shown that treatment with IL-21+ low-dose IL-2 significantly delayed early tumor growth, we compared the effects of the three different cytokine regimens on long-term tumor regression and overall survival beyond 30 days. Figure 2 shows individual tumor growth curves and long-term survival for all mice in each test group collected from three separate experiments. The large majority of tumor-bearing mice that received sublethal irradiation only (Figure 2A) had progressing tumors ≥ 200 mm^2 ^by day 28. Control animals that received sublethal irradiation and the hgp100_25–33 _vaccine exhibited a short delay in tumor progression, but >80% of the mice had tumors ≥ 200 mm^2 ^by day 35. There were no long-term survivors among mice whose treatment did not include a cytokine (Figure 2A, B). Approximately half of the mice treated with low dose IL-2 demonstrated a modest delay of tumor growth to ≥ 200 mm^2 ^until approximately day 42, and there was one long-term survivor (Figure 2C). IL-21 treatment resulted in an even longer delay (Figure 2D) in tumor progression, with 50% of animals reaching day 49 with tumors < 200 mm^2^, and 3/15 mice from the three experiments survived long-term. Mice treated with both IL-21 and IL-2 exhibited the best anti-tumor response. Among 24 mice from three separate experiments approximately 63% survived to day 49 with tumors < 200 mm^2^, and 46% (11/24) remained tumor free 63 days after tumor inoculation (Figure 2E). The cumulative long-term survival of mice from all groups from three experiments followed out to 150 days post tumor inoculation showed that IL-21 + IL-2 treated mice had significantly better survival (46%) compared to all other groups (Figure 2F). Combined therapy with IL-21 and IL-2 resulted in improved survival compared to vaccination only (p << 0.0001), vaccination plus IL-2 (p << 0.0001), or vaccination plus IL-21 (p = 0.035). The IL-21 only effect was significantly better than the vaccine only control (p = 0.030), but there was no significant difference between the long-term survival response to IL-21 alone and IL-2 alone (p = 0.80). All surviving mice in the IL-21 + IL-2 test group were tumor-free at day 150-post tumor inoculation.

### Tumor-Specific Protective Immunity in Long-Term Survivor Mice

**Figure 3:**
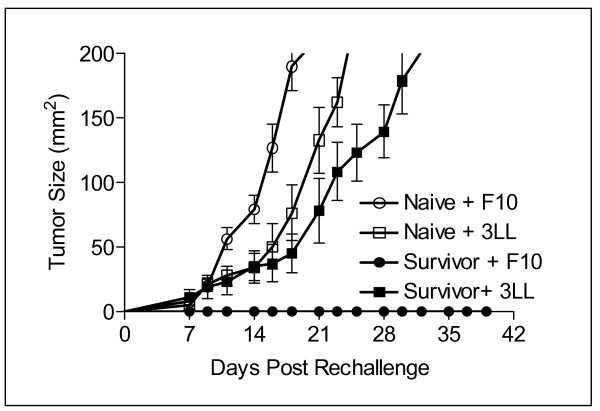
Tumor-specific protective immunity in long-term survivor mice. IL-21 + IL-2 treated mice that survived disease free for 150 days were rechallenged with B16F10 (5 × 10^5^) or 3LL(5 × 10^4^) tumor cells. All 3LL challenged mice (4/4) exhibited mean tumor growth to ≥ 200 mm^2 ^by day 32; all B16F10 challenged mice (5/5) were protected. Naïve mice challenged with the same B16F10 (5/5) or 3LL (5/5) tumor cells had rapid mean tumor growth to ≥ 200 mm^2 ^by day 25.

To determine whether long-term tumor-specific protective immunity developed after treatment with IL-21 + IL-2, mice surviving tumor free for 150 days were subsequently rechallenged subcutaneously with 5 × 10^5 ^B16F10 tumor cells or 5 × 10^4 ^3LL tumor cells. Tumor size was monitored three times per week out to 39 days (Figure 3). All 4 mice injected with control 3LL cells developed tumor (mean size >150 mm^2^) by day 32, while all 5 mice challenged with B16F10 were tumor-free to day 39, and remained so for another 150 days (data not shown). Additional control groups included naive congenic C57BL/6 mice inoculated with B16F10 (5 mice) or 3LL (5 mice) tumor cells. The mean tumor size for both tumors in naive control mice reached 200 mm^2 ^before day 25. Thus, the regimen of adoptive cell transfer with lymphopenic conditioning and tumor antigen vaccination followed by IL-21 + IL-2 therapy was curative in mice with established tumors, and produced a long-term tumor-specific memory response capable of protecting against a secondary challenge with the original tumor.

### IL-21 + IL-2 therapy induced high numbers of circulating pmel CD8^+ ^T cells

**Figure 4:**
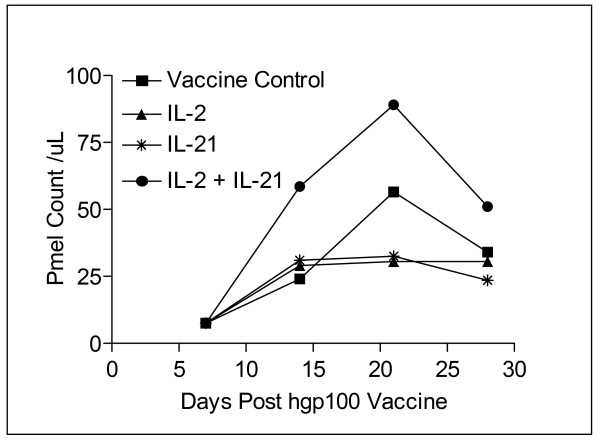
Quantitation of absolute numbers of pmel CD8^+ ^T cells. Pooled blood from mice in the vaccine control and all three cytokine experimental groups (≥ 5 mice/group) was analyzed by flow cytometry to determine the absolute number of circulating pmel-1 CD8^+ ^T cells/μL of blood. Vaccinated mice treated with IL-21+ IL-2 had the highest total pmel CD8^+ ^cell numbers at all time points. Data are shown from a representative experiment and show that IL-21 + IL-2 resulted in significantly higher circulating pmel CD8^+ ^T cell numbers than was produced by IL-2 (p = 0.05), or IL-21 (p = 0.05) alone.

Representative data from one of three experiments demonstrated that IL-21 + IL-2 therapy in the pmel Tg/B16F10 model induces higher absolute numbers of circulating pmel CD8^+ ^T cells than either IL-2 or IL-21 therapy alone (Figure 4). Pooled blood from 5–10 mice in each cytokine group and the vaccine only control were stained for CD8/CD127/CD62L/CD45.2 expression, and the absolute number of circulating pmel CD8^+ ^T cells was determined using counting beads as described. Analysis of variance (ANOVA) was performed on cell counts made at weekly intervals from 7–28 days post vaccine administration from pooled samples for each group; there was a significant difference between test groups (p = 0.029). A follow-up (post hoc) Tukey test showed that the absolute number of circulating pmel CD8^+ ^T cells in peripheral blood was significantly higher after combined cytokine therapy than following IL-2 (p < 0.05), or IL-21 (p < 0.05) therapy alone at 14, 21, and 28 days after vaccination. The expansion of circulating pmel cells appeared to peak at day 21 and go through a contraction phase from day 21 to day 28 in mice treated with IL-21+ IL-2, and for mice in the vaccine only control group. Cell numbers remained relatively constant from day 14–28 for mice treated with IL-21 or IL-2 alone.

### Cell surface phenotype of pmel CD8^+ ^T cells from cytokine-treated mice

**Figure 5:**
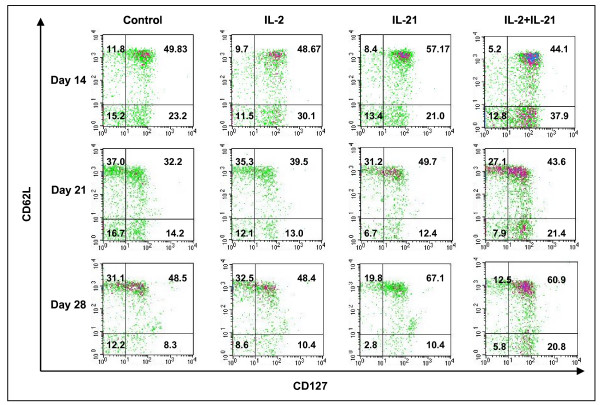
CD27 vs CD62L 2 parameter phenotype analysis of pmel CD8^+ ^T cells. Lymphocytes from pooled blood (5–10 mice/group) in a representative experiment were analyzed for CD127 and CD62L staining. Flow cytometry phenotype analysis of pmel CD8^+ ^T cells on days 14, 21 and 28 post vaccination indicated higher frequencies of antigen specific Tcm (CD62L^+^/CD127^+^) were induced at all time points by IL-21 alone compared to the control and IL-2 stimulated PBMCs; IL-21 + IL-2 stimulated comparably high Tcm frequencies on days 21 and 28. IL-21+IL-2 stimulated the highest frequency of pmel CD8^+ ^Tem (CD62L^-^/CD127^+^) at all time points.

Detailed pmel CD8^+ ^T cell subset analysis (CD8/CD127/CD62L/CD45.2) was performed on pooled peripheral blood samples from mice used in the experiment described in Figure 4. Recent reports have identified CD127 (IL-7α receptor) expression as an important descriptor for long-lived memory T cells which may arise early after in vivo priming [29-34]. These studies also indicated that CD62L and CD127 staining partitioned antigen-specific cells into two distinct memory T cell subsets which, by functional criteria, were similar to central memory T cells (Tcm) with a CD62L^+^/CD127^+ ^phenotype, and effector memory (Tem) cells with a CD62L^-^/CD127^+ ^phenotype [32,33]. Effector T cells (Te) were CD62L^-^/CD127^- ^and were characterized by poor proliferation response, immediate effector function, and high levels of antigen-induced apoptosis [33]. Figure 5 shows dot plots of CD127 versus CD62L staining of pre-gated CD8^+^/CD45.2^+ ^peripheral blood pmel T cells obtained on days 14, 21 and 28 from each cytokine therapy group and vaccine only control mice. The frequency of circulating pmel CD8^+ ^T cells with a Tcm phenotype on day 14 was no different in IL-2, and IL-21 + IL-2 treated mice or in the vaccine controls. Only IL-21 treated mice showed a small increase in Tcm frequency, which was 15% higher than that produced in control mice. Tem frequencies following treatment with IL-2 or with IL-21 + IL-2 were 30% and 63% higher respectively than in control mice. The frequencies of both Tcm and Tem pmel CD8^+ ^T cells decreased on day 21 for all experimental groups compared to day 14 values. The day 21 frequency of Tem pmel CD8^+ ^T cells (CD62L^-^/CD127^+^) was similar for all test groups except IL-21 + IL-2, which maintained approximately a 70% higher Tem percentage. The frequencies of Tcm (CD62L^+^/CD127^+^) on day 21 indicated IL-2 sustained a slightly higher percentage than the control (22%); IL-21 stimulation resulted in a 54% higher frequency of Tcm CD8^+ ^T cells compared to the control, and IL-21 + IL-2 treated mice maintained a Tcm frequency 35% higher than the control. By day 28 the control and IL-2 induced Tcm frequencies were equal to each other but higher than on day 21, while the IL-21 effect (38% increase) and the IL-21 + IL-2 effect (25% increase) on Tcm expression were both similarly elevated compared to the control and IL-2 induced percentages. Combined IL-21 and low-dose IL-2 therapy sustained the circulating Tem frequency at a level that was 2-fold higher than the control group or the effect induced by IL-2 alone or IL-21 alone. The cell density plots for IL-21+IL-2 on all three days also suggested an overall higher absolute number of circulating Tcm and Tem CD8^+^/CD45.2^+ ^T cells than was induced by any other treatment. Generally, lower percentages of circulating effector cells (CD62L^-^/CD127^-^) were present at day 21 and day 28 for IL-21 and IL-21+IL-2 treated mice compared to both IL-2 treated animals and control mice. The upper left quadrant of each 2-parameter histogram contained CD62L^+^/CD127^- ^T cells, and their frequency was generally lower in the IL-21 and IL-21+IL-2 treated animals. Presently there is no correlated functional data to determine the lineage relationship of this population to Tcm or Tem T cells or their functional properties. Overall the data in Figure 5 suggest IL-21+IL-2 induced higher frequencies of Tem cells compared to IL-2 or IL-21 alone on all three days; and by the early contraction phase of the pmel CD8^+ ^T cell response (day 28) IL-21 and IL-21 + low-dose IL-2 induced equally higher percentages of tumor-specific Tcm cells than were stimulated in the control or IL-2 treated mice.

### IL-21+ IL-2 induced proliferation of Tem and Tcm pmel CD8^+ ^T cells

**Figure 6:**
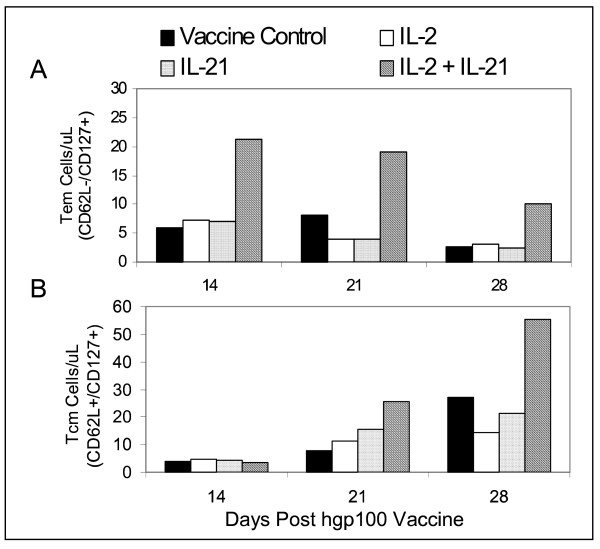
Quantitation of absolute numbers of Tem and Tcm CD8^+ ^T cells. PBMCs were examined by flow cytometry to determine the absolute number of Tem (CD62L^-^/CD127^+^) pmel CD8^+ ^T cells (A), and Tcm (CD62L^+^/CD127^+^) pmel CD8^+ ^T cells (B) on days 14, 21, and 28 after vaccination and cytokine treatment. IL-21 + IL-2 supported the highest level of circulating Tem cells on all three days compared to all other experimental groups (p = 0.011), and stimulated the greatest increase of Tcm cells on days 21 and 28 (p = 0.029). Data was collected from pooled blood from ≥ 5 mice in each test group.

The frequencies of Tcm and Tem T cells on days 14, 21 and 28 (Figure 5), and the total number of circulating pmel cells determined for each group (Figure 4) were subsequently used to calculate the absolute number of Tem (Figure 6A) and Tcm (Figure 6B) pmel CD8^+ ^T cells for each experimental group. Tem numbers on each day were significantly higher for IL-21+ IL-2 treated mice than for all other groups (p = 0.011) as determined by ANCOVA, and declined from day 14 to day 28 (Figure 6A). By contrast, the absolute number of Tcm cells for all groups was equally low at day 14 and increased incrementally for each group over time (Figure 6B). IL-21 + IL-2 induced Tcm cell numbers increased at a significantly higher rate compared to all other test groups (p = 0.029) as determined by ANCOVA. Thus, IL-21+ IL-2 treatment maintained Tem absolute numbers at all time points that were 2- to 4-fold greater than those produced by the vaccine control or by IL-2 or IL-21 therapy alone. Similarly, Tcm absolute numbers induced by IL-21+ IL-2 were 2- to 4-fold higher than those stimulated in any other test group by day 28. Recent data have demonstrated that the adoptive transfer of pmel CD8^+ ^Tcm (characterized as CD62L^+^/CCR7^+^), or "early effector" T cells (CD62L^dim^/CD127^+^/CCR7^+^) produced a potent anti-tumor response subsequent to tumor-antigen hgp100_25–33 _vaccination and high-dose IL-2 therapy in the pmel Tg/B16 therapeutic model [22,35]. The data in Figure 6B suggest IL-21+ low-dose IL-2 in vivo therapy promotes the long-term maintenance of CD8^+ ^T cells with a similar Tcm (CD62L^+^/CD127^+^) or "early effector" – like phenotype (CD62L^dim/-^/CD127^+^).

### Functional phenotype of CD8^+ ^T cells from IL-21 + IL-2 treated tumor regressor mice

**Figure 7:**
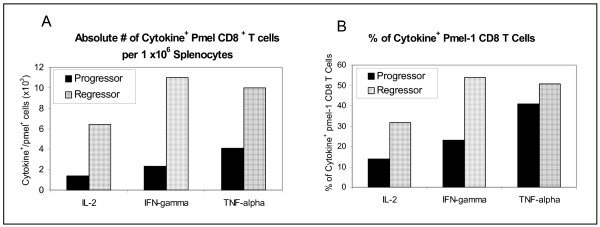
Cytokine flow cytometry analysis of the functional phenotype of pmel CD8^+ ^splenocytes from IL-21 + IL-2 treated mice. Spleen cells were pooled from 2 mice with progressing tumors (≥ 50 mm^2^) and from 2 mice with regressing tumors (<10 mm^2^) 42 days after vaccination. Cytokine flow cytometry analysis shows the absolute number of pmel CD8^+ ^T cells per 10^6 ^splenocytes (A), and the frequency of pmel CD8^+ ^cells (B) that are positive for IL-2, IFNγ, and TNFγ production. Data show mice with regressing tumors had 4.7-fold, 4.8-fold and 2.5-fold more IL-2^+^, IFN-γ^+^, and TNF-α^+ ^pmel CD8^+ ^T cells respectively, and higher frequencies of Tc1 cytokine^+ ^cells than progressor mice.

By day 42 previously regressing tumors in a subset of IL-21 + IL-2 treated mice began to grow again, reaching a mean tumor size of 25–50 mm^2^. All tumors of this size on day 42–49 continued to progress to ≥ 200 mm^2 ^by day 63, whereupon the mice were sacrificed. However, approximately 50% of all IL-21+ IL-2 treated mice in three experiments did not exhibit tumor growth, and by day 42–49 manifested complete regression of the tumor. These animals remained tumor free out to >150 days. Day 42 splenocytes from two "progressor", and from two "regressor" mice treated with IL-21 + IL-2 were pooled separately, and were stimulated in vitro with hgp100_25–33_; cells were then analyzed for IFNγ, IL-2 and TNF-α production using a standard five-hour CFC assay. After IVS cells were fixed, permeablized, and stained with CD8, CD45.2 and cytokine-specific antibodies. Spleens from regressor mice had more IFNγ, IL-2 and TNF-α positive CD8^+^/CD45.2^+ ^pmel T cells than spleens from progressor animals (Figure 7A). Regressor mice had 4.7-fold, 4.8-fold, and 2.5-fold more IL-2^+^, IFNγ^+ ^and TNF-α^+ ^pmel CD8^+ ^T cells, respectively, than progressor mice with rapidly growing tumors. The data in Figure 7A indicates that tumor regression in mice receiving IL-21+ IL-2 therapy was associated with an increase in the absolute number of pmel CD8^+ ^T cells producing Tc1 cytokines compared to the numbers found in mice with growing tumors. The presence of higher numbers of Tc1 cytokine^+ ^CD8^+ ^T cells in regressor compared to progressor mice was attributable to both a 2-fold greater absolute number of pmel CD8^+ ^T cells in regressor mice than were measured in progressor animals (data not shown) and to higher frequencies of pmel CD8^+ ^T cells which were positive for Tc1 cytokines in regressor mice at 42 days (Figure 7B).

## Discussion and conclusions

We report for the first time the anti-tumor effects of a treatment strategy that combines lymphopenic conditioning, adoptive transfer of naive antigen-specific CD8^+ ^T cells, tumor antigen-specific vaccination, and cytokine treatment with IL-21 + low-dose IL-2. Using a well established murine model of pre-existing disease, the pmel Tg/B16 model, our results show that combined IL-21 + low-dose IL-2 therapy delayed B16F10 melanoma growth in the majority of mice, and resulted in a significant increase in tumor-free survival (46%) out to ≥ 150 days compared to mice treated with IL-21 or IL-2 alone. Surviving mice exhibited tumor-specific protective immunity since they resisted B16F10 rechallenge but succumbed to the unrelated 3LL tumor. These data are in contrast to other studies in the pmel Tg/B16 model, which employed the adoptive transfer of highly activated IVS pmel CD8^+ ^T cells (stimulated with hgp100_25–33 _peptide and IL-2 or IL-15) rather than naive pmel T cells [3,17,18]. After lymphopenic conditioning and T-cell transfer to tumor-bearing mice, therapy was administered in these studies using different combinations of hgp100_25–33 _vaccination and cytokine treatment. Thus, Lou et al [17] vaccinated with hgp100_25–33 _peptide-pulsed DCs, and administered high-dose IL-2 (1.2 × 10^6 ^IU/day) for three days beginning the day of vaccination. Klebanoff, et al [18] vaccinated with a recombinant fowl pox virus encoding hgp100_25–33 _(rFPhgp100) after adoptive transfer of IVS pmel CD8^+ ^T cells grown in IL-2 or in IL-15 cultures. Follow-on cytokine therapy consisted of high-dose IL-2 (1.2 × 10^6 ^IU/day) for three days. In the only published study using IL-21 in the pmel Tg/B16 model Zeng and coworkers [3] also vaccinated with rFPhgp100_25–33 _following adoptive transfer of IL-2 cultured pmel T cells, and animals were treated over three days with IL-15 (20 μg/day), IL-21 (20 μg/day), or both cytokines at the same doses. None of these treatment strategies resulted in long-term inhibition of tumor growth beyond 30–35 days after treatment unless very large numbers (4–6 × 10^6^) of IVS pmel CD8^+ ^T cells were adoptively transferred [17]. Furthermore, in contrast to our results, none of these studies presented data describing long-term tumor-free survival ≥ 150 days with any combination of transferred T cells, vaccine administration and cytokine therapy. Another group compared the ability of IL-2, IL-15 or IL-21 to augment anti-tumor immunity in C57BL/6 mice challenged with the OVA-expressing E.G7 thymoma [6]. Cytokine therapy was initiated 48 hours after tumor inoculation – well before the establishment of vascularized tumors, and was administered every other day for two weeks thereafter. In this model IL-21 (20 μg/day) did result in prolonged tumor-free survival out to 100 days in approximately 25–30% of treated mice. Neither low-dose IL-2 (2 × 10^3 ^IU and 20 × 10^3 ^IU/day) nor IL-15 (5 μg and 50 μg/day) produced a survival frequency that was as high. All animals that survived beyond 100 days were protected from rechallenge with E.G7 tumor cells. Importantly, this report also showed that the 2-week cytokine administration could begin as late as day 12 following tumor inoculation and still produce a therapeutic response [6]. This study also demonstrated the ability of IL-21 to augment immunity to the OVA-expressing E.G7 tumor in the absence of previously antigen-educated CD8^+ ^T cells. However, the experimental design did not test the anti-tumor effects of IL-21 in a model of established bulky disease such as the pmel Tg/B16 system, in which the cognate tumor antigen is a relatively weak self-tumor antigen rather than a strongly immunogenic foreign protein. To date only the combined therapeutic effects of IL-21+ low-dose IL-2 reported here have resulted in long-term survival of tumor-free mice in the pmel Tg/B16 model. As noted, in most of the previous studies using the pmel Tg/B16 melanoma model very high doses (1.2 × 10^6 ^IU/day) of IL-2 were used in combination with the adoptive transfer of large numbers (up to 10^7^) of highly activated IVS pmel CD8^+ ^T cells [16-18] However, treatment was initiated after very large, bulky (50–100 mm^2^) tumors were established. By contrast, cytokine therapy was initiated in our experiments when vascularized tumors were smaller (26 mm^2^). Long-term survival using our current IL-21 + IL-2 therapeutic model may decrease with larger established tumors; experiments designed to test and optimize IL-21 +low-dose IL-2 therapy in mice with larger vascularized tumors are ongoing.

The H-2D^b^-restricted NV peptide was used in all experiments as a putative CD4 "helper" antigen source [23,24] However, our recent experiments suggest NV peptide vaccination produces only a modest increase in circulating pmel CD8^+ ^T cell numbers over that produced by hgp100_25–33 _alone (unpublished data – H.M. Hu, EACRI). CD4 helper T cell function in our DC-based vaccine system may have been provided by fetal calf serum (FCS) proteins/peptides associated with the DCs in the vaccine – both DCs and the B16F10 melanoma cells used in the tumor inoculum were cultured in medium containing FCS. The potential for FCS antigen-induced CD4 T cell activation is also suggested by other hgp100_25–33_-pulsed DC vaccine studies in the pmel Tg/B16 model in which anti-tumor therapeutic effect was achieved in the absence of any known source of CD4-specific antigen [17].

There is limited information on IL-21-mediated changes in the cell surface and functional phenotype of CD8^+ ^T-cells associated with tumor immunity. In this report, we show that IL-21 alone and IL-21+ low-dose IL-2 increased the in vivo frequency of Tcm T cells as defined by concomitant CD62L^+^/CD127^+ ^staining[29], compared to the vaccine only control or to cells from IL-2-treated mice (Figure 5). IL-21 + IL-2 treatment also sustained the highest percentage of Tem (CD62L^-^/CD127^+^) pmel CD8^+ ^T cells [29] compared to any other test group. This observation correlated with the result that IL-21+ IL-2 therapy also produced the highest absolute number of circulating pmel CD8^+ ^T cells during the beginning (day 14), peak (day 21) and end (day 28) of the hgp100_25–33 _induced expansion of pmel CD8^+ ^T cells (Figure 4). As a consequence, IL-21 + IL-2 therapy produced the highest absolute number of circulating pmel CD8^+ ^Tem cells at each time point of the expansion and early contraction phases of the anti-hgp100_25–33 _immune response, and also resulted in the highest absolute number of Tcm T cells in the peripheral blood (Figure 6). Although Tem absolute numbers decreased from day 14 to day 28, and Tcm numbers increased over this same period the data do not indicate if this is attributable in anyway to innate differences in IL-21 + IL-2 induced Tem vs Tcm proliferation. Future in vitro and in vivo studies will examine the relative proliferative potential of cognate antigen-driven purified populations of pmel CD8^+ ^Tcm and Tem cells. Notably, the tumor-specific protective memory response observed in long-term survivor mice was also associated with high frequencies of Tcm in the spleen and lymph nodes. Polychromatic (8 color) flow cytometry analysis of pmel CD8^+ ^splenocytes from IL-21 + IL-2 treated long-term survivor mice (> 150 days) indicated approximately 10%–12% of all CD8^+ ^T cells in the spleen and lymph nodes were pmel CD8^+ ^T cells, and 43% and 70% of these T cells in the lymph nodes and spleen respectively expressed a Tcm phenotype (CD62L^+^/CD127^+^/CD27^+^/CD28^+^)(unpublished data). Recent data have demonstrated that enriched pmel CD8^+ ^Tcm T cells conferred a potent in vivo anti-tumor recall response upon adoptive transfer – leading to the eradication of large established tumors in the pmel Tg/B16 model [35]. Thus, as described herein, IL-21 + IL-2 therapy favored the in vivo expansion and maintenance of such tumor antigen-specific Tcm T cells from naive pmel CD8^+ ^T cell precursors which were capable of providing long-term protective immunity. Preliminary phenotype analysis of IL-21 + low-dose IL-2 and hgp100_25–33 _IVS pmel splenocytes similarly indicates the combination of both cytokines results in the proliferation of pmel Tg CD8^+ ^T cells with a Tcm (CD62L^+^/CD127^+^/CD27^+^/CD28^+^) phenotype (unpublished data – EACRI). This further suggests that IL-21 + low-dose IL-2 might be effective in supporting the cognate antigen-driven in vitro expansion of rare self-tumor antigen Tcm CD8^+ ^T cells from autologous PBMCs – perhaps in concert with procedures to remove tumor antigen-specific regulatory T cell effects. In addition to increasing tumor-antigen directed expansion and maintenance of Tcm CD8^+ ^T cells, data presented here demonstrates that IL-21 + IL-2 therapy also stimulated the expression of anti-tumor memory CD8^+ ^T cells with a Tc1 cytokine functional phenotype in mice with regressing tumors. By day 42 post-tumor inoculation IL-21 + IL-2 treated tumor-regressor mice had much higher absolute numbers of IL-2^+^, IFNγ^+ ^and TNF-α^+ ^pmel CD8^+ ^splenocytes than IL-21 + IL-2 treated tumor-progressor mice with rapidly growing tumors (Figure 7). The relative absence of CD8^+ ^splenocytes with a Tc1 cytokine functional phenotype in tumor progressor mice may be attributable in part to a simple lack of sustained B16F10 tumor antigen stimulation. Previous published data from our institute using the pmel Tg/B16 model has demonstrated loss of MHC class I and gp100 expression in a high percentage of tumors in experimental animals with progressive disease due to immunoediting[36]. Overall our data suggest that IL-21 + low-dose IL-2-mediated tumor regression and long-term survival was associated with elevated absolute numbers of tumor antigen-specific Tem and Tcm CD8^+ ^T cells with a Tc1 functional phenotype.

IL-21 + low-dose IL-2 induction of increased in vivo expression of Tc1 cytokine^+ ^Tcm and Tem CD8^+ ^T cells is a central observation for any putative mechanistic explanation of the anti-tumor therapeutic synergy produced using both cytokines. This observation examined in the context of recent studies describing the broad inhibitory effects of IL-21 on B lymphocytes, NK cells and DCs (Reviewed in [37]) suggests a possible mechanism for the IL-21+ low-dose IL-2 induced expansion of Tcm CD8^+ ^T cells similar to what may occur during the late contraction phase of an ongoing immune response. Thus, CD4 T cell (Th2)-derived IL-21[38] inhibits anti-IgM and IL-4-mediated B cell proliferation [2] and enhances apoptosis of activated B cells [39,40], inhibits DC maturation and the ability of DCs to prime T cells [41,42]; and increases NK maturation and cytolytic function while inhibiting NK proliferation [43]. Similarly, while IL-21 has generally been described as an inducer of antigen stimulated IFNγ and CTL function in CD8^+ ^T cells [4,8,44,45], our unpublished data in both murine and human in vitro experiments indicate IL-21 alone does not drive high levels of cognate antigen stimulated CTL proliferation. In vivo data presented here suggest that IL-21 works best in concert with low-dose IL-2 to support both optimal anti-tumor effector CTL function and expansion of CD8^+ ^memory T cells. Paradoxically, IL-21 + low-dose IL-2 treatment produced unexpectedly low frequencies of circulating effector pmel CD8^+ ^T cells (Figure 4) – perhaps due to cell trafficking to the primary tumor and sites of metastatic disease. Ongoing experiments are directed at the in situ analysis of the phenotype, cell number, and functional properties of tumor invasive pmel CD8^+ ^T cells in IL-21 + low dose IL-2 treated mice. Such studies should shed light on whether combined cytokine therapy induces trafficking of increased numbers of antigen-specific cytolytic CD8^+ ^T cells to the tumor site. Taken together, these observations suggest a dual role for IL-21 in dampening ongoing innate and adaptive immune responses (as might occur during the contraction phase of antigen-specific immunity), while concomitantly augmenting the expansion of antigen-driven long-term memory T cells. Recently published data demonstrate IL-21 treated cultures of antigen-stimulated cells produce CD8^+ ^T cells with increased TCR binding affinity [5]. Other data show the IL-21 receptor (IL-21R) is upregulated upon TCR engagement [46,47]. Thus, Th2-derived IL-21 may "rescue" memory T cells by blocking further antigen-driven effector T cell differentiation through presently unknown mechanisms, while simultaneously increasing antigen binding affinity. The resulting increase in binding of low-levels of residual antigen (present during the contraction phase) could increase IL-21R expression and up-regulate increased IL-21 binding to memory T cells – thus further skewing the shift to memory T cells. Memory CD8^+ ^T cells in turn express amplified levels of CD122 [48], which may facilitate increased high affinity IL-2R formation if other IL-2R chains (CD25 and CD132) are available, and potentially increase binding of the low levels of IL-2 which may be present during immunological contraction. The combination of the "arrested" Tcm memory phenotype (maintained by IL-21), and low dose IL-2-induced proliferation of these cells could result in increased Tcm cell numbers. Thus, IL-21 + low-dose IL-2 therapy may favor the enhanced expansion of long-term memory (Tcm) CD8^+ ^T cells through augmented memory T cell "rescue" and expansion mechanisms similar to those which may normally be present during the late contraction phase of an ongoing immune response. This concept is supported by the results of our study and other reports [6,8], which suggest IL-21 induced tumor immunity is most effective when IL-21 is administered several days (4–12 days) after initial tumor-antigen activation of T cells. The late acting anti-tumor effects of IL-21, and, in our study, IL-21 + IL-2 therapy suggests IL-21 may be acting directly on tumor antigen educated CD8^+ ^T cells rather than modulating early APC and/or T cell function associated with primary tumor antigen stimulation. Preliminary in vitro studies with purified naïve and hgp100_25–33 _stimulated pmel CD8^+ ^T cells support this conclusion (data not shown).

In summary, the results of this study suggest that IL-21 + low-dose IL-2 cytokine therapy may "rescue" and augment proliferation of tumor antigen-specific memory CD8^+ ^T cells, and thus provide an important new strategic component for effective cancer immunotherapy. The combined use of both cytokines may be most effective in the context of lymphopenic conditioning, tumor antigen-specific vaccination, and the adoptive transfer of autologous tumor-specific Tcm or "early" effector T cells. Ongoing work at our institute continues to focus on developing procedures for the IL-21 + low-dose IL-2-directed in vivo and in vitro expansion and maintenance of such Tcm and "early" effector subpopulations from small numbers of normally tolerized autologous self-tumor antigen-specific precursors.

## Competing interests

Christopher Clegg is a scientist employed by Zymogenetics – all other authors declare that they have no competing interests.

## Authors' contributions

HH designed the in vivo experiments and worked with PW and DH to perform the flow cytometry analysis of PBMC and splenocyte samples. GY assisted in all aspects of tumor inoculation, lymphopenic conditioning, tumor measurement and tissue harvest. HMH, CHC, and AOH provided key reagents and assisted in the design of controls which utilized these reagents in the experiments. WM was directly involved in drafting and revising the manuscript, and WGA performed all the statistical analysis. EBW designed and supervised all aspects of the experimental strategy and acquisition of data, and, with BAF and WJU, was involved in data interpretation and the critical review and revision of the manuscript.
